# Lactate-induced mtDNA Accumulation Activates cGAS-STING Signaling and the Inflammatory Response in Sjögren's Syndrome

**DOI:** 10.7150/ijms.83801

**Published:** 2023-08-15

**Authors:** Jiabao Xu, Changyu Chen, Junhao Yin, Jiayao Fu, Xiujuan Yang, Baoli Wang, Chuangqi Yu, Lingyan Zheng, Zhiyuan Zhang

**Affiliations:** 1 Department of Oral and Maxillofacial-Head Neck Oncology, Ninth People's Hospital, Shanghai Jiao Tong University School of Medicine, Shanghai, China.; 2 National Clinical Research Center for Oral Diseases, National Center for Stomatology, Shanghai, China.; 3 Shanghai Key Laboratory of Stomatology, Shanghai, China.; 4 Laboratory of Oral Microbiota and Systematic Diseases, Shanghai Ninth People's Hospital, College of Stomatology, Shanghai Jiao Tong University School of Medicine, Shanghai, China.; 5 Shanghai Research Institute of Stomatology, Shanghai, China.; 6 Research Unit of Oral and Maxillofacial Regenerative Medicine, Chinese Academy of Medical Sciences, Shanghai, China.; 7 Department of Oral Surgery, Ninth People's Hosptial, Shanghai Jiao Tong University School of Medicine, Shanghai, China.

**Keywords:** Lactate, mtDNA, Sjögren's Syndrome, Sodium Dichloroacetate, Salivary acinar cells

## Abstract

Acinar epithelial cell atrophy in secretory glands is a hallmark of primary Sjögren's syndrome (pSS), the cause of which is far from elucidated. We examined the role of acinar atrophy by focusing on the metabolism of glandular epithelial cells and mitochondria in the pSS environment. After confirming the presence of a high-lactate environment in the labial glands of human pSS patients, we used the A253 cell line and NOD/Ltj mice as models to investigate the metabolic changes in salivary gland epithelial cells in a high-lactate environment *in vitro* and *in vivo*. We found that epithelial cells produced high levels of IL-6, IL-8, IFN-α, IFN-β and TNF-α and exhibited significant NF-κB and type I IFN-related pathway activation. The results confirmed that lactate damaged mitochondrial DNA (mtDNA) and led to its leakage, which subsequently activated the cGAS-STING pathway. Inflammatory cytokine production and pathway activation were inhibited *in vivo* and *in vitro* by the lactate scavenger sodium dichloroacetate (DCA). Our study provides new insights into the etiology and treatment of pSS from the perspective of cell metabolism.

## Introduction

Sjögren's syndrome (SS) is a systemic autoimmune disease that primarily affects the exocrine glands (mainly the salivary and lacrimal glands), resulting in severe dryness of mucosal surfaces. The disease may occur only in exocrine glands, or it may occur in the context of lupus erythematosus (SLE) and other autoimmune diseases. The incidence of SS varies between 0.5% and 1% of the general population and is relatively high among all autoimmune diseases 1. SS can lead to B-cell tumors, which cause disproportionate mortality. This disease mainly affects middle-aged women but can also affect children, men and elderly individuals. When SS occurs in a previously healthy person, the syndrome is classified as primary SS (pSS). SS associated with another underlying systemic autoimmune disease, such as SLE, rheumatoid arthritis (RA), or scleroderma, is called secondary SS. The cause of SS is unknown, but both genetic and environmental factors are assumed to play roles 2.

As an autoimmune disease, SS is characterized by the common features of autoimmune diseases: lymphocyte infiltration and attack the body's organs and tissues. During this process, several pivotal metabolic processes, especially glucose metabolism, fatty acid metabolism and amino acid metabolism, are significantly elevated in CD4+ T cells, thereby satisfying the increased demand for energy, and these cells then secrete proinflammatory cytokines to mediate the innate or adaptive immune response. Our previous study suggested that several metabolic processes, such as glycolysis and glutaminolysis, are dramatically involved in the pathogenesis of SS 3. The metabolic products consequently accumulate in the infiltrated gland tissues. Among these, lactate, a well-studied final metabolite of glucose metabolism, has recently been considered an active molecule that can modulate the immune response, in addition to acting as an end metabolic product. Although lactate can be reutilized in muscle and the liver via the Cori cycle, it is primarily viewed as a byproduct of metabolism or as a biomarker in critical care rather than a bioactive molecule, and its functional effects have long been overlooked. Lactate accumulation in the microenvironment is far from inert and has a major impact on tissue-resident and infiltrating immune cells, and during inflammatory diseases, lactate accumulation in the tissue microenvironment can amplify inflammation 4-6. Excessive lactate accumulates at the sites of immune cell accumulation, leading to metabolic reprogramming of immune cells and exacerbating the development of inflammation. Studies have shown that lactate is significantly increased in the joint cavity in autoimmune diseases such as RA 7. Therefore, we hypothesized that the hypermetabolic lymphocytes of SS patients may cause abnormal lactate metabolism in salivary glands infiltrated by lymphocytes, which may elucidate the etiology of this autoimmune disease and aid in the development of a cure.

One of the unexplored correlates of the inflammatory etiology of autoimmune diseases is the specific contribution of organelles 8. In particular, mitochondria, which are key organelles for the survival of aerobic eukaryotic cells, are the main source of ATP, the regulator of the redox state and the main source of reactive oxygen species (ROS) and have also received increasing attention in recent years 9. Mitochondrial biogenesis and mitophagy are two processes that regulate mitochondrial levels and maintain organelle homeostasis 10. Strong regulation between these two opposing processes is critical for adaptation to cellular metabolic states, stress and other environmental or intracellular signals. An imbalance in biogenesis and mitophagy leads to the progressive development of pathological conditions associated with mitochondrial dysfunction, resulting in fatigue and other symptoms of most chronic diseases 11. Transmission electron microscopy revealed mitochondrial alterations in salivary gland epithelial cells from patients with SS, including swelling of the mitochondrial matrix, loss and disorganization of cristae, disruption of mitochondrial membranes, and myelin-like patterns within mitochondria 12. These changes can lead to the exudation of mitochondrial contents. Recent studies have shown that mitochondrial damage can trigger immune activation in both immune and nonimmune cells and that certain leaked components can act as damage-associated molecular patterns (DAMPs) and trigger immune responses. Circular mitochondrial DNA (mtDNA), which is structurally characterized by hypomethylated CpG motifs similar to prokaryotes, lacks histones, and contains fewer introns and polycistrons than nuclear DNA (nDNA) 13. Unlike nDNA, mtDNA is continuously replicated and transcribed throughout the cell cycle. During these processes, the bulk of mtDNA may be single-stranded for long periods of time and have a strong tendency to form noncanonical structures 14.

In the present study, we found the accumulation of lactate in the salivary glands of patients with SS. Accumulated lactate can damage and induce mitochondrial leakage in glandular epithelial cells, and the antigenic component mtDNA triggers a series of inflammatory responses, including nuclear factor kappa-B (NF-κB) inflammatory signaling, through the cyclic GMP-AMP synthase (cGAS) and interferon-stimulated gene (STING) pathways and the type I IFN response induced by interferon regulatory factor 3 (IRF3.) We also observed that glandular epithelial cell secretion can promote the proliferation of T lymphocytes and reduce apoptosis.

## Results

### Lactate levels are elevated in the salivary glands of patients with SS, and the transcript levels of inflammatory factors are increased

**Figure 1:**
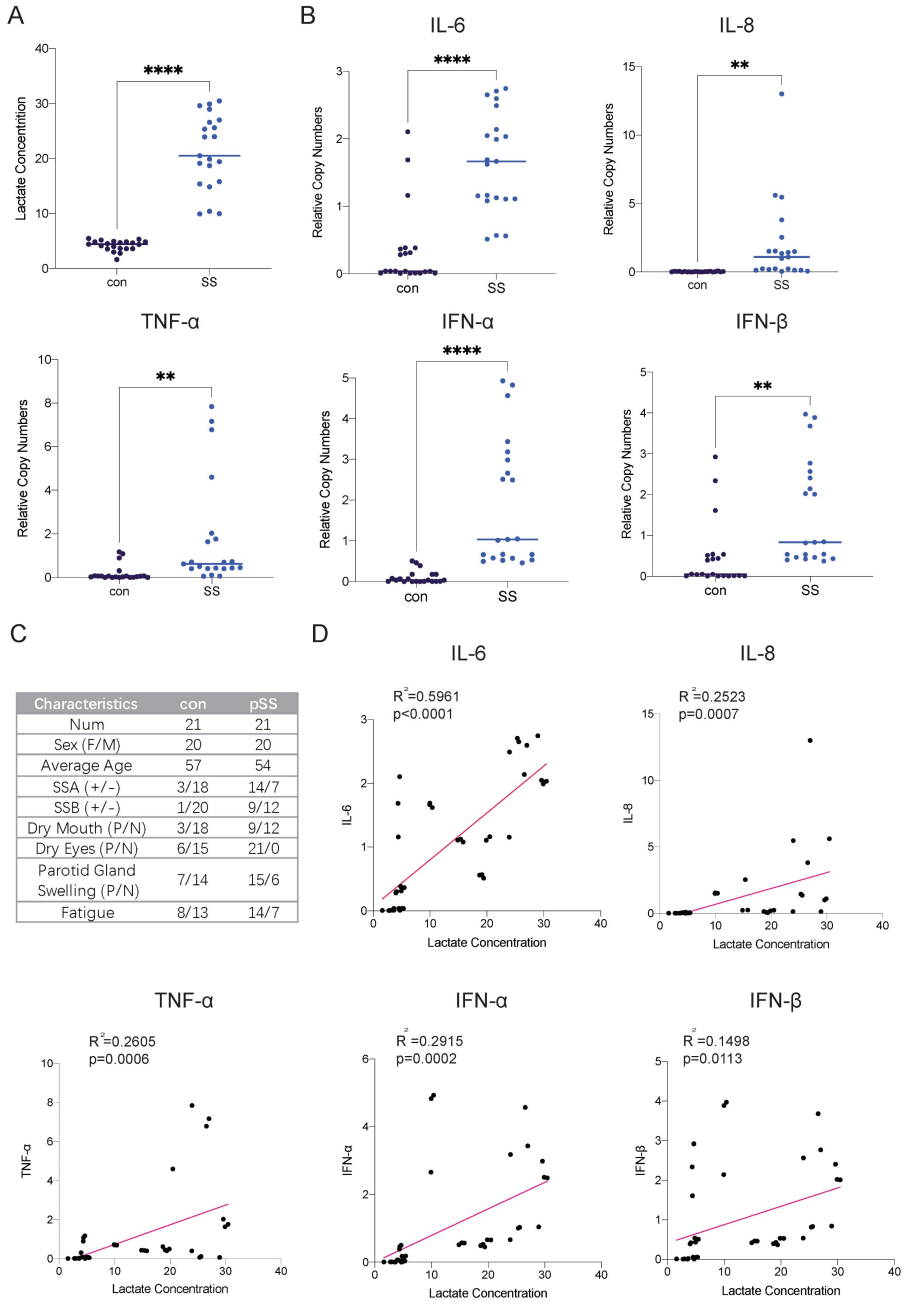
Elevated lactate levels in the labial glands of pSS patients and their correlation with cytokine expression. (A) Lactate concentrations in labial gland samples from healthy controls and patients with pSS. (B) RT-qPCR analysis of cytokine expression levels in labial gland samples from healthy controls and pSS patients. (C) Patient information and statistics of labial gland samples used in this study. (D) Correlation analysis between lactate concentrations and cytokine expression levels in labial gland samples.

Autoimmune diseases mostly manifest as chronic inflammation. Long-term lymphocyte infiltration produces a large number of inflammatory factors and affects the microenvironment of glandular cells 15. As the end product of glycolysis, lactate is often enriched in areas where cellular metabolism is active, such as inflammatory cell infiltration foci. Studies have shown that patients with RA have many infiltrating lymphocytes in their joint cavities and increased levels of lactate, but there is currently a lack of relevant research on SS 16. Therefore, we collected labial gland samples from SS patients and healthy controls and compared the lactate levels in the glands. We found that lactate levels in the labial glands were much higher in SS patients than in controls (Fig. 1A). The transcript levels of the inflammatory factors interleukin (IL)-6, IL-8, interferon (IFN)-α, IFN-β and tumor necrosis factor-α (TNF-α) were also much higher than those in normal subjects (Fig. 1B). Next, we conducted a correlation analysis of lactate levels and cytokine expression in the samples and found that glandular lactate concentrations were positively correlated with the transcript levels of inflammatory factors (Fig. 1D). Thus, we hypothesized that a high-lactate environment in the gland may exacerbate the progression of SS.

### A high-lactate environment leads to cellular NF-κB activation and a type I IFN inflammatory response

**Figure 2:**
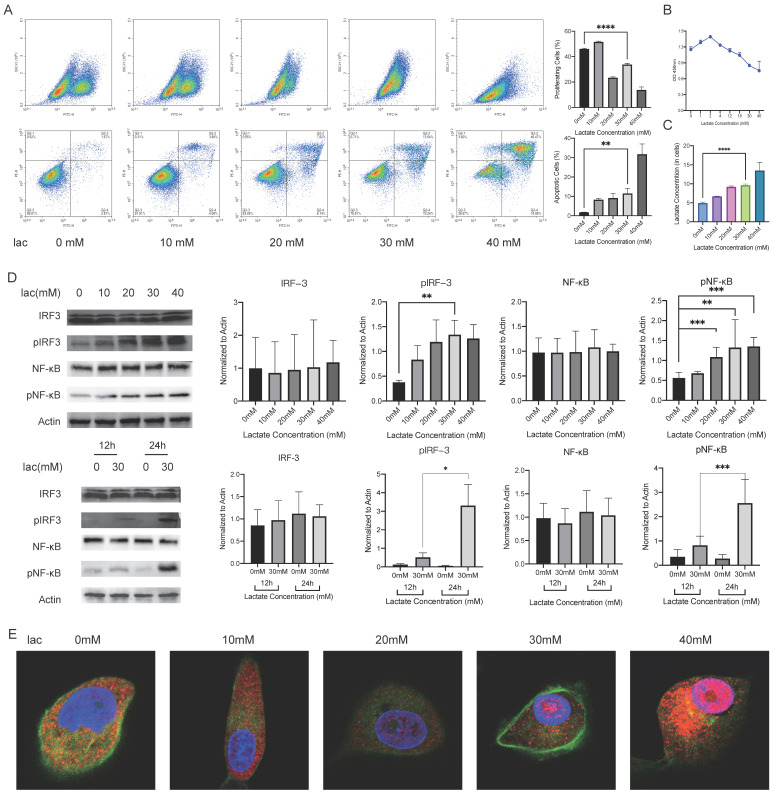
NF-κB activation and type I IFN responses in A253 cells activated by a high-lactate environment. (A, B) EdU, FITC-PE and CCK-8 staining showed changes in proliferation, apoptosis and the cell cycle of A253 cells after 24 h of incubation in medium supplemented with different concentrations of lactate. (C) Changes in intracellular lactate levels in response to different lactate concentrations. (D) Western blot analysis of the expression of IL-6, IL-8, TNFα, IFN-α and IFN-β upstream pathways in A253 cells treated with different lactate concentrations for different times. (E) Immunofluorescence shows the extent of NF-κB penetration into the nucleus.

We observed that the increased inflammatory factors in glands were effector inflammatory factors associated with NF-κB-mediated inflammatory responses, as well as type I IFN inflammatory responses. Next, we selected an appropriate cell model to examine the mechanism. Since the HSG cell line was contaminated with HeLa cells, we noted that in S Lisi et al., the submandibular gland tumor cell line A253 was used as an *in vitro* model to study SS. The results showed that this cell line has the same sensitivity to these autoantibodies (including anti-M3 Peptide IgG, Anti-RO and Anti-La, etc.) as other non-cancer cell lines. 17-21. When A253 cells were in lactate-supplemented culture, this cell line exhibited a similar disease progression as salivary gland acinar cells in patients with SS, including decreased proliferation, increased apoptosis, and changes in the cell cycle 20 (Fig. 2A, 2B). Furthermore, the change trend of intracellular lactate concentration in this cell line was similar to that in labial gland samples (Fig. 2C). These data suggest that the A253 cell line is a suitable research model. Subsequently, we measured the phosphorylation levels of NF-κB and IRF3, which are the upstream regulators of inflammatory factors such as IFN-α, IFN-β, IL-6, IL-8, TNF-α, in the presence of different concentrations of lactate for different times. The phosphorylation levels of NF-κB and IRF3 increased correspondingly with the same treatments (Fig. 2D). After being activated, NF-κB can enter the nucleus to regulate gene expression, and so we observed the nuclear entry of NF-κB by immunofluorescence staining. Consistent with our expectations, NF-κB migrated significantly into the nucleus when lactate was increased (Fig. 2E). These *in vitro* experiments demonstrated that a high-lactate environment could activate NF-κB and the inflammatory response of type I IFN.

### Lactate triggers an inflammatory response by damaging mtDNA and causing mtDNA leakage but not ROS production

**Figure 3:**
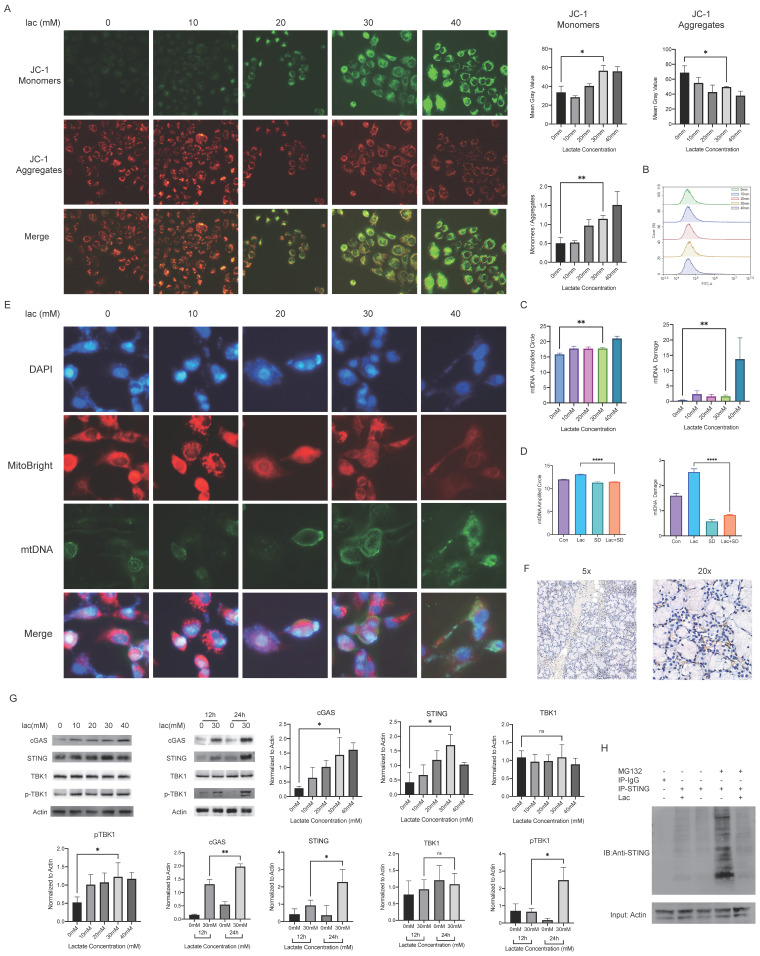
Mitochondrial damage and mtDNA damage leakage in cells in a high-lactate environment. (A) JC-1 staining was used to observe mitochondrial damage. (B) ROS changes in cells stained with DCFH-DA, indicating negative results. (C) mtDNA copy number and damage of A253 cells incubated with lactate. (D) mtDNA copy number and damage of A253 cells in 30 mM lactate, 15 mM DCA or both for 24 h. (E) Immunofluorescence staining of mtDNA; red indicates mitochondria, and blue (DAPI) indicates DNA. (F) Immunohistochemistry showing STING expression in the labial glands of human pSS patients. (G) Western blot analysis of the expression of free DNA receptors, IFN and NF-κB upstream signaling pathway in A253 cells. (H) IP was used to verify the degree of STING ubiquitination in 30 mM lactate environment.

In cells, lactate is commonly transformed into pyruvate in the cytoplasm and enters the mitochondria and is transformed into acetyl-CoA to enter the tricarboxylic acid cycle. As an energy substrate and a potential signaling molecule, lactate can promote cellular mitochondrial metabolism and energy production to a certain extent, but a sustained increase in lactate metabolism can lead to mitochondrial stress 22. Mitochondria are the center of cellular energy. Once mitochondria are damaged, the energy metabolism of cells will be seriously disturbed, eventually leading to cell death. Therefore, we were very interested in whether the mitochondria of epithelial cells were damaged in a high-lactate environment and examined the mitochondrial membrane potential using JC-1 staining; the results showed that mitochondrial membrane damage was exacerbated with increasing lactate concentrations (Fig. 3A). Byproducts of normal mitochondrial metabolism and homeostasis include the accumulation of potentially damaging ROS and Ca^2+^. Since mitochondria are the center of cellular aerobic respiration, the most common result of abnormal aerobic respiration is an increase in ROS production 23. Studies have shown that lactate can lead to oxidative stress and mitochondrial damage by promoting ROS production 24. We therefore tested the effect of lactate on ROS production in A253 cells, but unfortunately, we did not find significant changes in ROS levels (Fig. 3B). This sparked our curiosity, and we started exploring other possibilities. Due to the similarity of mitochondria to prokaryotic cells, we realized that mitochondrial damage resulting in altered properties and the leakage of contents could also trigger an immune response. Leakage components, which are termed DAMPs, are recognized by many receptors, including the TLR family of receptors, and lead to downstream inflammatory responses 25. Therefore, we measured mtDNA damage, which is the most common mitochondrial antigen, by qPCR and found that mtDNA damage increased with increasing lactate concentrations (Fig. 3C). To confirm that lactate was responsible for this change, we used the lactate scavenger sodium dichloroacetate (DCA, 15 mM) in the presence of lactate and found that DCA significantly reduced mtDNA damage (Fig. 3D). Subsequently, we determined the leakage of mtDNA by immunofluorescence staining (Fig. 3E).

The cGAS-STING pathway is known to recognize cell-free DNA in the cytoplasm, whether this DNA comes from viruses, bacteria or their own mitochondria 26. Moreover, TANK-binding kinase 1 (TBK1) is also involved in the activation of both STING and NF-κB 27. Because c-GAS is rapidly degraded after production and recognized by STING, we conducted immunohistochemical staining of STING on the labial gland sections of pSS patients, and the results showed that STING was not only greatly enriched around the infiltration foci of T cells but also accumulated around the epithelial cells (Fig. 3F) 26. We measured the expression level of cGAS-STING and the phosphorylation level of TBK1 in cells by Western blotting and found that they were increased in a high-lactate environment (Fig. 3F). Furthermore, we treated the cells in a high-lactate environment for different times and found that the expression of these factors increased with increasing treatment times (Fig. 3G). This result indicated that the lactate-enriched environment caused mtDNA damage and that the cGAS response required a certain period of time.

Since cGAS is easily degraded, we hypothesized that the duration of STING activity may be a key factor in the subsequent series of inflammatory responses. In normal organisms, to avoid an excessive immune response, STING is often degraded by ubiquitination after activation 28. We identified the promotion of STING expression in a high-lactate environment, and so we examined whether this environment could affect the breakdown of STING. IP experiments showed that the ubiquitination-mediated degradation of STING was significantly reduced by lactate (Fig. 3H). Furthermore, we also found significant enrichment of STING by immunohistochemical staining of human labial gland sections (Fig. 3H). This finding strongly illustrates the contribution of lactate to the overactivation of this pathway. cGAS-STING is a component of innate immunity in humans, and its role in autoimmune diseases such as SS is extremely important. Finally, we attempted to use a Seahorse experiment to investigate whether a high-lactate environment affects the oxidative phosphorylation ability of cells. The results showed that the mitochondrial oxygen consumption rate (OCR) did not change meaningfully with lactate concentration (Supplement 1). In addition, ATP production capacity and spare respiratory capacity did not change significantly, suggesting that lactate did not affect cell metabolism through oxidative phosphorylation.

### Lactate is essential for the activation of cGAS-STING and its downstream pathways

**Figure 4:**
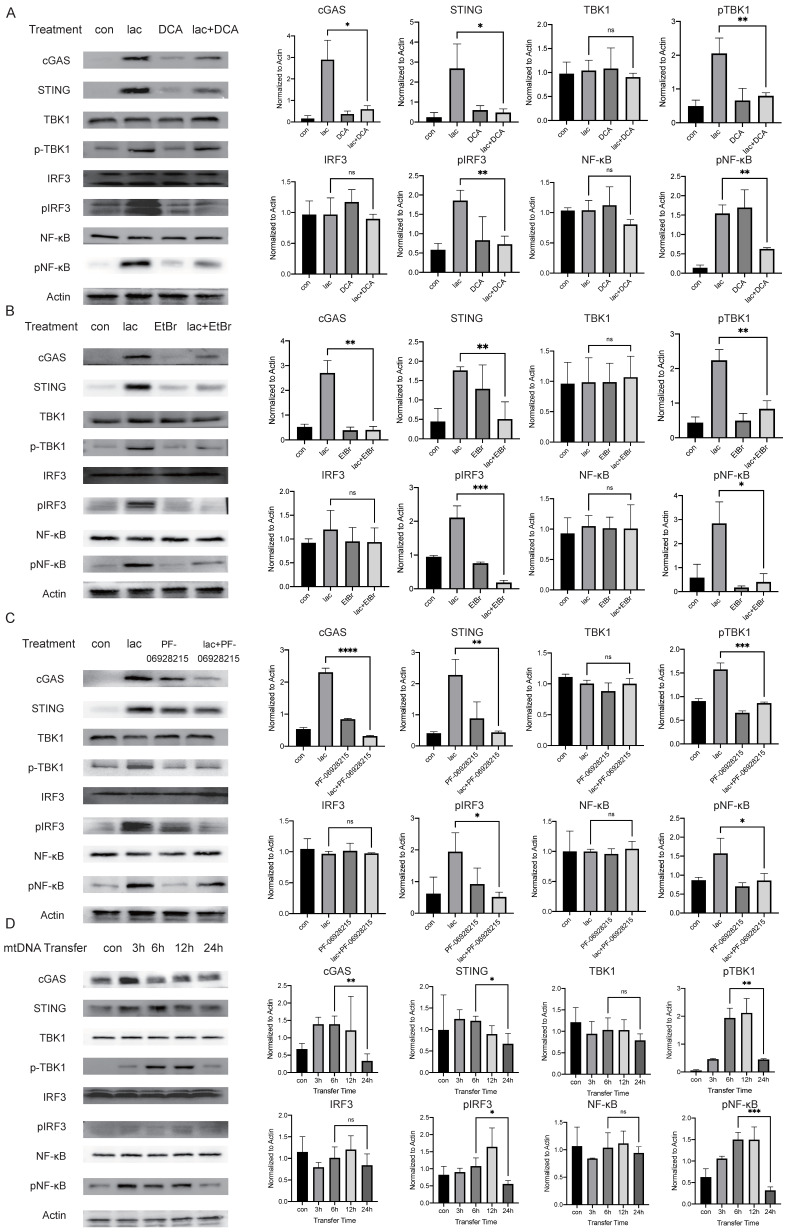
High lactate is necessary for mtDNA damage and subsequent pathway activation. (A) Western blot analysis showed that cGAS-STING and subsequent pathway activation in A253 cells were attenuated after incubation with 15 mM DCA for 24 h. (B) Western blot analysis showed that cGAS-STING and subsequent pathway activation in A253 cells were attenuated after incubation with 1 μg/mL EtBr for 48 h. (C) Western blot showing that after incubation with 4 mM PF-09628215 for 1 h before lactate treatment, the activation of cGAS-STING and subsequent pathways in A253 cells were weakened after addition of lactate for another 24 h. (D) Western blot analysis showed that after exogenous mtDNA was transferred into cells, the activation of cGAS-STING and subsequent pathways changed over time.

We performed a series of validations in cell lines to confirm our results. First, we incubated cells with lactate in the presence of DCA and found that DCA significantly reduced the pathway activation induced by lactate, illustrating the requirement of lactate in this effect (Fig. 4A). We do not deny that there may be more factors involved in the activation of cGAS in patients with SS. The etiology of SS is not clear; some studies suggest that the development of SS may have involve viruses, and the genetic material of viruses can also activate cGAS^29^. However, in this study, we focused more on the cause of autoimmunity, and we also successfully identified a major cause of cGAS activation. Subsequently, we examined whether mtDNA activated downstream pathways. Ethidium bromide (EtBr) has been used in several publications to deplete mtDNA with no effect on nDNA 30. Therefore, we incubated cells with low concentrations of EtBr for a longer period of time (1 μg/ml, 48 h) to deplete mtDNA in cells and found that the activation of the pathway was significantly reduced in cells treated with both EtBr and lactate (Fig. 4B) 31. Next, we demonstrated the requirement of cGAS in this reaction using an inhibitor of cGAS (Fig. 4C). Finally, we tried to transfer the ND1 gene fragment of the extracted and amplified mtDNA from the A253 cell line into normal A253 cells and harvested the cells at different times. We found that the expression of cGAS peaked 3 h after mtDNA transfer and then started to decrease slowly (Fig. 4D). This result shows that when cells are incubated with lactate, the small molecule needs time to enter the cell, act on mitochondria to cause mtDNA damage and leakage and activate cGAS in order to induce subsequent pathways. The activation of cGAS was followed by rapid inactivation of the protein, but subsequent pathway protein degradation was much slower than cGAS due to the reduction in STING ubiquitination (Fig. 4D).

### The lactate scavenger DCA inhibits disease progression in NOD/Ltj mice

**Figure 5:**
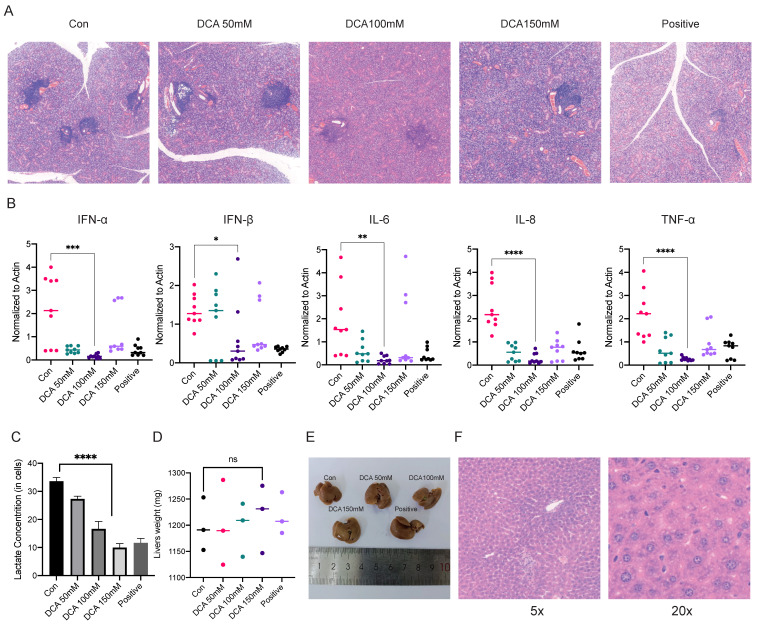
Administration of a lactate scavenger attenuates autoimmune responses in 8-week-old NOD/Ltj mice. (A) After DCA (50 mg/kg, 100 mg/kg, 150 mg/kg), prednisone acetate (65 mg/kg) and solvent treatment for 4 weeks, H&E-stained sections were obtained from the submandibular glands of the mice. (B, C) Comparison of inflammatory factor expression and lactate concentrations in the submandibular glands of mice treated with DCA (50 mg/kg, 100 mg/kg, 150 mg/kg), prednisone acetate (65 mg/kg) and solvent for 4 weeks. (D-F): Effects of drug treatment on liver texture, weight, and stem cells in mice.

In previous experiments, we examined the mechanism of lactate in a cellular model, and next, we used NOD/Ltj mice with autoimmune diseases to perform *in vivo* validation. We used 8-week-old female NOD/Ltj mice administered intraperitoneal injections of different concentrations of DCA on alternate days for a total of four weeks; prednisone solution was used as a positive control, and the mice were given the same treatment duration. It was obvious that DCA had an effect on the disease course of NOD/Ltj mice (Fig. 5A). We also examined the expression of inflammatory factors and the concentrations of lactate in the glands of the mice, which further confirmed that the clearance of lactate could reduce inflammation in the glands (Fig. 5B, C). Moreover, the injection of DCA did not cause damage to the livers of the mice (Fig. 5D-F).

### Epithelial cell secretions in a high-lactate environment can increase lymphocyte proliferation while reducing apoptosis

**Figure 6:**
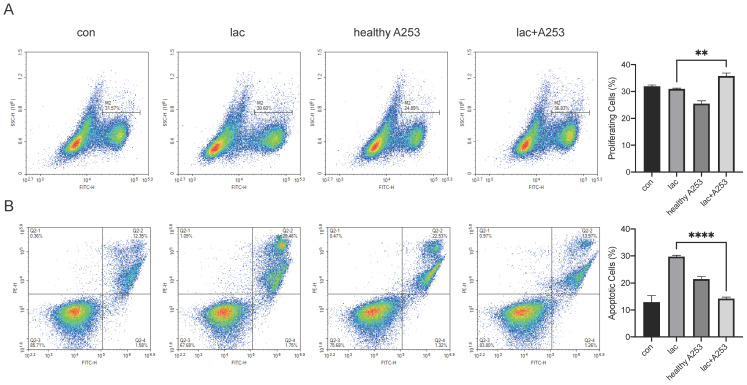
Changes in epithelial cells also caused changes in lymphocyte proliferation and apoptosis in the presence of high lactate. (A) EdU was used to detect changes in the proliferation of Jurkat cells caused by A253 cells. (B) FITC-PE staining showed apoptotic changes in the Jurkat cell line caused by A253 cells.

Due to the infiltration of a large number of lymphocytes in the gland, there are bound to be interactions between the cells. We modeled cell‒cell interactions *in vitro* using A253 cells and Jurkat cells. First, we incubated the A253 cell line with lactate and normal medium. After 24 h, Jurkat cells were divided into four groups and cultured in the following media: normal 1640 medium, 1640 medium with lactate, normal A253 cell-conditioned 1640 medium, and lactate-supplemented A253 cell-conditioned medium. After culture for 24 h, Jurkat cell proliferation and apoptosis were examined. We found that lactate-only medium increased the levels of apoptosis in Jurkat cells, and A253-only medium resulted in fewer changes in Jurkat cells than A253-conditioned medium, which significantly reduced Jurkat cell apoptosis and increased proliferation (Fig. 6A, B).

## Discussion

In summary, we found high concentrations of lactate in the labial glands of SS patients and confirmed that lactate causes mtDNA damage and leakage, which is recognized by cGAS-STING and activates NF-κB and type I IFN-mediated inflammation. We also found that epithelial cells in a high-lactate environment affected lymphocyte proliferation and apoptosis, further revealing the important role of epithelial cells in the progression of SS.

Recent studies have shown that the activation of innate immunity in salivary gland cells is involved in the occurrence and progression of pSS. Although the initial events that cause innate immune activation in the salivary gland epithelium remain to be determined, several possibilities have been proposed. These include the involvement of exogenous antigens or aberrant expression of endogenous factors (e.g., retrofactors) that stimulate innate immune responses. Another possibility is that these responses are triggered by DAMPs due to the inefficient removal of epithelial cell debris 32. Our study demonstrates the contribution of the DAMP mtDNA to the innate immune response in epithelial cells. Multiple important innate immune response pathways are activated by mtDNA, including the cGAS-STING pathway. As a research hotspot in recent years, this pathway has different characteristics from other innate immune pathways; its activator is an important part of life. Innate immune dysregulation, especially changes in intensity and duration, can trigger a variety of abnormal innate immune responses to disrupt homeostasis in the organism 33. At present, studies have confirmed that cGAS-STING and its downstream pathways cause IFN-I-type inflammatory responses in monocytes and dendritic cells of pSS patients and are also involved in the development of various autoimmune diseases, such as SLE, RA, and AGS. 34, 35. STING undergoes conformational changes mainly through self-palmitoylation, resulting in interactions with TBK1 and the phosphorylation of IRF3 and NF-κB 36. TBK1 is a critical node. First, STING recruits TBK1 and mediates IFN-dependent inflammatory responses through IRF3. Second, TBK1 suppresses inflammation by phosphorylating and inducing the degradation of the IKK kinase NIK, thereby attenuating NF-κB activity. However, increased TBK1 phosphorylation in pSS patients leads to increased NF-κB-mediated inflammatory responses 37-39. The use of TBK1 inhibitors can reduce the symptoms of a variety of autoimmune diseases 40. This further confirms the important role of cGAS-STING in the pathogenesis and progression of pSS by a series of cell-intrinsic innate autoimmune system abnormalities.

In our study, the accumulation of lactate and cGAS-STING were innovatively linked via mtDNA. This may be an important cause of pSS symptoms. STING, which is a key signal transduction molecule involved in innate immune responses, is triggered by cytoplasmic DNA from pathogens and hosts to induce the secretion of type I interferons and proinflammatory cytokines and defends against viruses and intracellular bacteria. STING plays an important role in infection and the regulation of spontaneous antitumor immune responses *in vivo*. When the DNA sensor cGAS detects cytoplasmic double-stranded DNA (dsDNA) released by dead cells, tumor cells or pathogens and mitochondrial DNA (mtDNA) leaking into the cytoplasm, cGAS catalyzes ATP and GTP synthesis of the second messenger 2',3'-cGAMP and directly activates the STING protein in the endoplasmic reticulum. In addition, STING can also directly act as a DNA sensor, recognizing DNA carried by bacteria and triggering an innate immune response to pathogens. The aggregates formed by STING activation first recruit two main downstream molecules: TBK1 and IRF3. TBK1 phosphorylates IRF3, activates the IRF3 dimer to enter the nucleus, induces the expression of type I IFNs (such as IFN-β), and initiates the interferon immune response. In addition, the transcription factor NF-κB can also be activated by STING to enter the nucleus and induce the expression of proinflammatory cytokines (such as TNF-α), and IL-6. The most secreted cytokine in the STING pathway is IFN-β, which can not only directly kill cancer cells but also mediate antigen presentation by promoting the maturation of dendritic cells. Innate and adaptive immune responses are linked 41. In 2019, Zhang et al. used cryo-electron microscopy to determine how activated STING aggregates specifically recruited TBK1 and IRF3 to achieve TBK1-dependent phosphorylation of IRF3. It was found that a STING dimer binds a TBK1 dimer, and the TBK1-binding motif (TBM) at the end of the C-terminal tail (CTT) released by activating STING binds to the groove of the TBK1 dimer interface, thereby achieving the high-affinity recruitment of TBK1 by STING. In STING aggregates, multiple TBK1 dimers aggregate, and trans-autophosphorylation occurs between TBK1 dimers. While only some CTTs of STING dimers in the aggregates recruit TBK1, Ser366 adjacent to CTTs that do not bind TBK1 is phosphorylated by TBK1. The 363LXIS366 motif is phosphorylated by STING and constitutes an IRF3 binding motif, which recruits IRF3 to be phosphorylated by TBK1, activates IRF3 dimerization into the nucleus, and activates the type I IFN response 37.

In other studies, lactate is considered to be a key node in immune regulation. The blood lactate concentration in healthy individuals is stable at 1.5-3 mM 42. However, tumors and sites of inflammation, including immune diseases, are associated with lactate accumulation due to enhanced glycolysis. Lactate is produced and accumulates in the extracellular space due to hypoxia or aerobic glycolysis 43. In our study, the lactate concentration in normal human salivary glands was approximately 5 mM. This result suggests that the lactate level in the microenvironment of salivary glands is higher than that of the overall environment, such as blood. The lactate concentration in the labial glands of pSS patients can reach 30 mM, which is similar to that in the synovial fluid of RA patients; the lactate concentration in the tumor environment can reach 40 mM. The accumulation of lactate plays a broad role in immune system dysregulation in autoimmunity. Several studies on glucose metabolism in RA suggest that glycolytic enzymes are involved in autoimmune responses. Specifically, enolase, aldolase, glucose-6-phosphate isomerase, and adipokines (i.e., leptin and adiponectin) have been identified as mechanisms of self-tolerance disruption in peripheral blood and synovial tissue cells. Potential triggers that have been shown to induce immune cell activation, the secretion of proinflammatory cytokines (i.e., TNF-α, IL-17, IFN-γ) and the production of autoantibodies (i.e., anti-cyclic citrullinated peptides), leading to autoimmunity and bone destruction 44, 45. SLE CD4+ T cells exhibit metabolic dysregulation in both humans and mice. In particular, CD4+ T cells from SLE patients exhibit enhanced glycolysis and mitochondrial metabolism, which correlates with their activation state. Inhibition of these pathways (using 2-deoxyglucose and metformin, respectively) reduced IFN-γ production *in vitro* and normalized T-cell metabolism and the disease phenotype *in vivo*. Furthermore, mitochondrial membrane hyperpolarization increases the activity of mammalian target of rapamycin (mTOR) in CD4+ T cells from patients with SLE, and rapamycin treatment reduces disease manifestations in these patients 46-49. Increased extramitochondrial glucose metabolism in cerebrospinal fluid and serum was significantly higher in patients with multiple sclerosis (MS) than in controls, and these factors were positively and correlated with disease progression and activity 50-52. Our study showed that lactate concentrations in glands were positively correlated with the expression of inflammatory cytokines, that the lactate scavenger DCA significantly alleviated the progression of the NOD/Ltj mouse model, and that inhibiting glycolysis could inhibit glycolysis and attenuate CD4 (+) T-cell proliferation and SS-like autoimmune responses 3. These findings show the important roles of lactate in autoimmune diseases. Of course, high concentrations of lactate can also lead to immune changes in the tumor environment. In the presence of oxygen, tumor cells selectively utilize glycolysis, which is also known as aerobic glycolysis or the Warburg effect, and lactate produced in this environment has been shown to inhibit human CTL proliferation, cytokine production and migratory capacity in cancer patients 53, 54. This is different from the effect of lactate on immunity in autoimmune diseases. We hypothesize that this difference is due to the accumulated lactate concentration: the lactate concentration in autoimmune diseases is mildly high, while the lactate concentration associated with tumor immunity is excessively high. The mechanism is worthy of further examination.

In our study, we focused on one problem: mtDNA is normally damaged by ROS accumulation in mitochondria. However, in our study, no ROS changes were observed, and mitochondria were damaged in the high-lactate environment 55. Therefore, mtDNA is likely damaged by other means. The generation of ROS mainly depends on oxidative phosphorylation. The accumulated lactate can be transformed into pyruvate and converted into acetyl-CoA to enter the tricarboxylic acid cycle, which is decomposed by oxidative phosphorylation and produces a large amount of ROS 23. However, because the process of oxidative phosphorylation is limited by pyruvate dehydrogenase (PDH), simply increasing the concentration of lactate will not cause changes in PDH levels or overload oxidative phosphorylation 56. Our experiments also show this. The OCR level, ATP production level and spare respiratory capacity of A253 cells did not change meaningfully under the condition of only increasing lactic acid. Therefore, we assume that the retained lactate in cells leads to an increase in intracellular acidity and that the change in pH may also cause mitochondrial stress and mtDNA damage 57.

## Conclusions

In conclusion, our results reveal a novel possibility regarding pSS: lactate produced by immune cells causes mtDNA damage leakage by acting on mitochondria in acinus epithelial cells and activates NF-κB signaling through cGAS-STING recognition, which exacerbates the immune response of epithelial cells. Our study adds new insights into the etiology and treatment of pSS.

## Supplementary Material

Supplementary figure S1.

## Materials and Methods

### Tissue samples

Labial gland tissues from 15 SS patients (Fig. 1c) and control tissues from 15 healthy donors were obtained from the Department of Oral Surgery of Shanghai Ninth People's Hospital in 2021, and all patients signed informed consent forms. This study was approved by the Ethics Committee of Shanghai Ninth People's Hospital (Grant No. SH9H-2019-T159-2).

### Cell culture

The human submandibular tumor cell line A253 (American Type Culture Collection, ATCC, Maryland, USA) was cultured in complete high-glucose DMEM (11965092, Gibco, New York, USA). Human T-lymphoblastic (Jurkat) cells were purchased from The Cell Bank of China and cultured in RPMI 1640 (11875093, Gibco). The media contained 10% fetal calf serum (FBS, Gibco) and 1% penicillin/streptomycin (SV30010, HyClone, Utah, USA). The cells were incubated in a humidified incubator with 5% CO2 at 37°C. The cultured cells were used for experiments when they reached 60-70% confluence. When lactate incubation was performed, the cells were seeded on Petri dishes on the first day. After they adhered to the plate on the second day, 10 mM, 20 mM, 30 mM and 40 mM lactate was added and incubated for 24 h, and then the subsequent analyses were performed.

### Animal experiments

Female NOD/Ltj mice were purchased from the Model Animal Research Center of Nanjing University (China). For experiments involving sodium dichloroacetate (DCA, 347795-10G, Sigma‒Aldrich, Missouri, USA), prednisone acetate (HY-B1832, MCE, New Jersey, USA) was used as a positive control. Treatment was performed starting at the age of 8 weeks. The mice were divided into 5 groups of 5 mice each, and DCA (50 mg/kg, 100 mg/kg, 150 mg/kg), prednisone acetate (65 mg/kg) and solvent were administered by intraperitoneal injection every other day. For the dissolution of prednisone acetate, we referred to the recommended method on the MCE website. After four weeks of treatment, the mice were sacrificed, and salivary gland tissues were extracted. The tissues were fixed and embedded in paraffin for hematoxylin & eosin (H&E) staining. The severity of gland tissue lesions was estimated by a widely used scoring system [16,17] in which a lymphocytic focus was defined as a group of > 50 lymphocytes in a random field (4 mm^2^). Briefly, the scoring system classified 0 as no lymphocytic infiltration, 1 as slight lymphocytic infiltration, 2 as moderate lymphocytic infiltration of less than 50 lymphocytes, 3 as one lymphocytic focus, and 4 as more than one lymphocytic focus. In addition, the total lymphocytic foci containing a group of less than 50 lymphocytes was also counted in the sections. Each group contained at least 3 mice that were treated as indicated. During the experiment and routine feeding, animals received humane care according to the criteria outlined in the Guide for the Care and Use of Laboratory Animals published by the National Institutes of Health (NIH) [18]. Furthermore, we examined cytokine transcript and glandular lactate levels in the submandibular glands of the mice. This study was approved by the Committee of Ethics of the Shanghai Jiao Tong University Faculty of Medicine.

### Lactate measurement

After the cells were incubated in a high-lactate environment for 24 h, they were harvested and permeabilized with 300 μL of 0.1% Triton X-100. Then, according to the requirements of the lactate assay kit-WST (L256, Dojindo, Japan), the cells were centrifuged, and 20 μL of the supernatant was added to a 96-well plate. The diluted enzyme was added and incubated at 37°C for 30 min in the dark. A microplate reader (BioTek, Vermont, USA) was used to measure the optical density at 450 nm. The lactate assays in human labial gland tissue were similar to the cellular lactate assays. This entailed grinding a 2 mm3 labial gland sample in 300 μL of 0.1% Triton X-100, followed by centrifugation to collect the supernatant. Subsequently, 20 μL of this supernatant was transferred to a 96-well plate. Enzyme reagent was then added and the plate was incubated in a dark environment at 37°C for 30 minutes, before an absorbance measurement was taken using a microplate reader. The lactate concentration was determined by constructing a lactate standard curve, using the protocol indicated in the kit. Finally, absorbance was converted to actual lactate concentration of the solution.

### Cell viability assay

A Cell Counting Kit 8 (CCK‐8, C0038, Beyotime, China) assay was used to determine the effect of different concentrations of lactate on A253 cell viability. We plated A253 cells in a 96‐well plate and maintained their density at 3,000/well/100 μL for adherence for 24 h prior to adding lactate (BCCF8868, Sigma). The final concentrations of lactate were 0, 1, 2, 4, 12, 18, 30, and 40 mM. After 24 h, the cells were ready to be tested. Five replicate wells were set up in each sample group. Briefly, the medium was removed, and A253 cells were gently washed with phosphate‐buffered saline (PBS, BL302A, Biosharp, China) after each time period. Then, 10 μL of CCK‐8 solution and 90 μL of medium were added to each well and incubated for 1 h. A microplate reader (BioTek) was used to measure the optical density at 450 nm. Each assay was performed at least in triplicate.

### EdU assay

The EdU assay was used to detect the effect of lactate on the proliferation of A253 cells. After A253 cells were treated with different concentrations of lactate (0 mM, 10 mM, 20 mM, 30 mM, 40 mM) for 24 h, we incubated the cells with the EdU solution for 2 h. Then, we fixed the A253 cells with 4% paraformaldehyde for 15 min and stained them with a BeyoClick EdU‐555 kit (C0075S, Beyotime). Then, the cells were analyzed by flow cytometry (Agilent NovoCyte, California, USA) using Novo Express.

### RNA extraction and real‐time polymerase chain reaction (RT-PCR)

Total cellular RNA was extracted by using TRIzol reagent (15596018, Takara Bio, Japan). According to the manufacturer's instructions, we used a PrimeScript RT Reagent Kit (Perfect Real‐Time, RR037A, Takara Bio) to reverse-transcribe the RNA. Then, we used the cDNA obtained from 2 μg of RNA to perform RT-PCR with TB Green Premix Ex Taq (RR420A, TaKaRa Bio) on a LightCycler 480 II (Roche, Switzerland). We used LightCycler 480 SW 1.5.1 (Roche) to analyze the levels of RNA. All reactions were performed in triplicate in 384‐well PCR microplates, and β-actin was used as the internal control to normalize the gene expression levels. The primers were purchased from Sangon Biotech. First, the relative standard curve was used to evaluate the efficiency of the primers, and the fold change in RNA expression was calculated as -ΔΔCt. Then, quantitative results were obtained. The primers are listed in Table 1.

### Western blotting

Cells were treated with lactate (10 mM, 20 mM, 30 mM and 40 mM) and incubated in a 37°C incubator for 24 h. To investigate the effect of incubation time on the cells, we added 30 mM lactate and harvested cells at 6 h and 24 h. Total protein was collected using IP lysis buffer (UH290847, Thermo Fisher). The antibodies used are listed in Table 2. All antibodies were diluted 1:1000 according to the manufacturer's instructions. Then, the samples were subjected to standard Western blot procedures using a Bio-Rad system.

### Apoptosis analysis by Annexin V/PI assays

Cells were seeded in 6-well plates and treated with lactate (10 mM, 20 mM, 30 mM and 40 mM) for 24 h. An Annexin V/PI detection kit (PL748, Dojindo) was used according to the manufacturer's protocol. Briefly, the resuspended cells were collected in flow cytometry tubes and stained. Apoptotic cells were analyzed by flow cytometry.

### JC-1-based mitochondrial dysfunction analysis

JC-1 MitoMP Detection Kits (MT09, Dojindo) were used to evaluate mitochondrial function in A253 cells. Briefly, 2 μM JC-1 working solution was added 45 min prior to collection. After being treated with JC-1, the cells were washed twice with PBS and then imaged. The fluorescence strength was evaluated under a microscope.

### mtDNA isolation, damage analysis and transformation

After A253 cells were treated with lactate for 24 h, they were digested with trypsin (0.25% Trypsin-EDTA, 25200-056, Gibco), and total DNA was extracted with a TIANamp Genomic DNA Kit (#W0220). Quantitative PCR (qPCR) analysis of DNA damage was based on the principle that many kinds of DNA lesions can block or slow the progression of DNA polymerase. Therefore, if equal amounts of DNA from differently treated samples were amplified by qPCR under identical conditions, DNA with more lesions would amplify to a lesser extent than less damaged DNA. The quantification of DNA damage was performed by qPCR. mtDNA damage was quantified by comparing the relative efficiency of the amplification of mtDNA fragments (mtND1) of DNA to controls and normalizing this to nuclear DNA (B2M), which had a statistically negligible likelihood of containing damaged bases. All the primers were purchased from Sangon.

RMECs were transfected with mtDNA using Lipofectamine 3000 (L3000015, Thermo Fisher, Massachusetts, USA) according to the manufacturer's instructions. Briefly, the cells were incubated under normal growth conditions (37 °C and 5% CO2) and were 70% confluent on the day of transfection. The mtDNA was diluted in TE buffer, and 3.75 µl Lipofectamine 3000, 5 µl P3000 and 5 µg mtDNA were added to each well of a six-well plate and incubated for 13 min in advance. After 4 h of incubation, the cells were washed, the medium was changed, and the samples were collected at 1 h, 3 h, 6 h, and 24 h.

### H&E staining and immunohistochemistry

Paraffin wax-embedded sections (4 μm) were stained with H&E or used for immunohistochemistry. The antibodies used are listed in Table 2. The samples were incubated at room temperature with 3% H2O2 for 5-10 min to eliminate the activity of endogenous peroxidase. The cells were blocked with 5~10% normal goat serum and incubated at room temperature for 10 min. Biotin-labeled secondary antibodies diluted to the appropriate proportions were added dropwise and incubated at 37°C for 10-30 min. Then, horseradish peroxidase-labeled streptavidin in the appropriate proportion was added dropwise and incubated at 37°C for 10-30 min.

### ROS assay

A253 cells were seeded in six-well plates and cultured overnight until they adhered to the plate, and then lactate was added. After 24 h, the cell culture medium was replaced with serum-free medium containing 1:1000 DCFH-DA (S0033S, Beyotime), and the cells were cultured at 37°C for 20 min, washed three times with serum-free medium, and digested with trypsin to prepare a cell suspension for analysis by flow cytometry.

### Immunoprecipitation

A253 cells were treated with lactate, DCA, and sodium lactate with DCA for 24 h, and total protein was extracted. Ten microliters of each sample were taken out as input, and the rest was combined with IP magnetic beads (UG288056A, Thermo Fisher Scientific) and STING antibody. The next day, the samples were boiled for 10 min. Then, the samples were subjected to standard Western blot procedures using a Bio-Rad system.

### mtDNA damage staining

A253 cells were treated with different concentrations of lactate for 24 hours, and mitochondria were stained with a mitochondrial staining kit (MT15, Dojindo) for 30 minutes. DNA was fixed with 4% paraformaldehyde and stained with DAPI for 10 min. Finally, the samples were observed under fluorescence microscopy.

### Metabolic flux assay

The cellular OCR in the A253 cells was analyzed on an XF96 Extracellular Flux Analyzer (Seahorse Bioscience, Massachusetts, USA). Prior to the experiment, 2 × 10^5^ cells (per well) were plated on Seahorse 96-well culture plates precoated with Cell-Tak (DLW354240, Corning). The extracellular acidification rate (ECAR) and OCR were measured in XF DMEM containing glutamine, glucose, HEPES and pyruvate under basal conditions and in response to oligomycin, carbonyl cyanide-p-trifluoromethoxyphenylhydrazone (FCCP), rotenone and antimycin A at the concentrations specified in the instructions.

### Statistical analysis

The data were analyzed using SPSS 26.0 and GraphPad Prism 9. Paired samples t tests were conducted to compare parameters between groups. All statistical tests were two-sided, and the level of statistical significance was set at p < 0.05.

## Figures and Tables

**Table 1:** Primers

Gene	Forward Primer	Reverse Primer
ND1	CACTTTCCACACAGACATCA	TGGTTAGGCTGGTGTTAGGG
B2M	GAGGCTATCCAGCGTACTCCA	CGGCAGGCATACTCATCTTTT
β-actin	CTCCATCCTGGCCTCGCTGT	GCTGTCACCTTCACCGTTCC
TNFA	CCTCTCTCTAATCAGCCCTCTG	GAGGACCTGGGAGTAGATGAG
IL-6	ACTCACCTCTTCAGAACGAATTG	CCATCTTTGGAAGGTTCAGGTTG
IFNA	GCCTCGCCCTTTGCTTTACT	CTGTGGGTCTCAGGGAGATCA
IFNB	ATGACCAACAAGTGTCTCCTCC	GGAATCCAAGCAAGTTGTAGCTC
tnf	CAGGCGGTGCCTATGTCTC	CGATCACCCCGAAGTTCAGTAG
il6	CTGCAAGAGACTTCCATCCAG	AGTGGTATAGACAGGTCTGTTGG
ifnb1	AGCTCCAAGAAAGGACGAACA	GCCCTGTAGGTGAGGTTGAT
ifna	GCAACCCTCCTAGACTCATTCT	CCAGCAGGGCGTCTTCCT

**Table 2:** Antibodies

Antibody	Source	Identifier
NF-κB p65 (D14E12)	Cell Signaling Technology	#8242
Phospho-NF-κB p65 (Ser536) (93H1)	Cell Signaling Technology	#3033
TBK1/NAK (D1B4)	Cell Signaling Technology	#3504
Phospho-TBK1/NAK (Ser172)	Cell Signaling Technology	#5483
IRF-3 (D83B9)	Cell Signaling Technology	#4302
Phospho-IRF-3 (Ser396) (D6O1M)	Cell Signaling Technology	#29047
β-Actin (D6A8)	Cell Signaling Technology	#8457
cGAS (E5V3W)	Cell Signaling Technology	#79978
STING (D2P2F)	Cell Signaling Technology	#13647
Anti-DNA antibody, double-stranded, clone BV16-13	Sigma	MAB030
HRP-labeled goat anti-rabbit IgG(H+L)	Beyotime	A0208
FITC-labeled goat anti-mouse IgG (H+L)	Beyotime	A0568

## Abbreviations

pSS: primary Sjögren's syndrome
SLE: systemic lupus erythematosus
RA: rheumatoid arthritis
ROS: reactive oxygen species
DAMPs: damage-associated molecular patterns
mtDNA: mitochondrial DNA
NF-κB: nuclear factor kappa-B
cGAS: cyclic GMP-AMP synthase
STING: stimulator of interferon genes
IL: interleukin
IFN: interferon
TNF: tumor necrosis factor
TBK1: TANK-binding kinase 1
