# Supplementary material for: Lactate-induced mtDNA Accumulation Activates cGAS-STING Signaling and the Inflammatory Response in Sjögren's Syndrome
Source: Int J Med Sci. 2023 Aug 15;20(10):1256–71. doi: 10.7150/ijms.83801 (PMC10542019; doi:10.7150/ijms.83801)
Supplement: Supplementary file 1 — Supplementary figure S1. [file ijmsv20p1256s1.pdf]

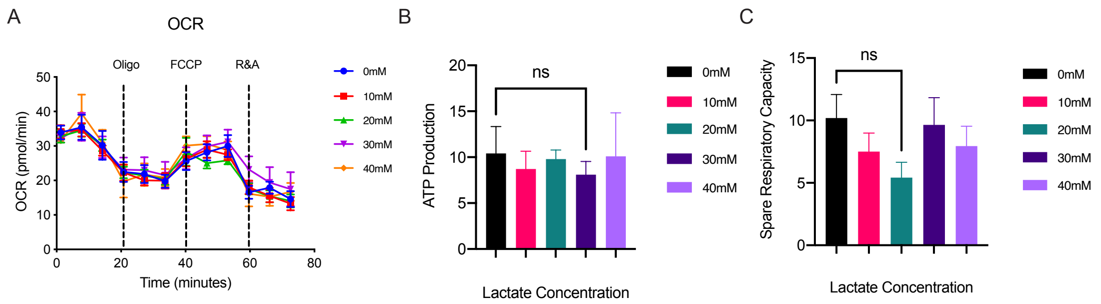

Figure S1 Effects of lactate on oxidative phosphorylation levels of A253. (A) A Seahorse assay was performed to measure OCR in A253 cells under different concentrations of lactate. (B, C) ATP production and spare respiratory capacity of A253 cells in a high-lactate environment.
